# Vaccine Effectiveness of Cell-Based Quadrivalent Influenza Vaccine in Children: A Narrative Review

**DOI:** 10.3390/vaccines11101594

**Published:** 2023-10-15

**Authors:** Joaquin F. Mould-Quevedo, Stephen I. Pelton, Van Hung Nguyen

**Affiliations:** 1 Seqirus USA Inc., Summit, NJ 07901, USA; 2 Chobanian and Avedesian School of Medicine, Boston University, Boston, MA 02118, USA; spelton@bu.edu; 3 VHN Consulting Inc., Montreal, QC H2V3L8, Canada

**Keywords:** QIVc, cell-based, influenza vaccine, pediatric, children, effectiveness, cost-effectiveness, hospitalization, medical encounters

## Abstract

Cell-based manufacturing of seasonal influenza vaccines eliminates the risk of egg-adaptation of candidate vaccine viruses, potentially increasing vaccine effectiveness (VE). We present an overview of published data reporting the VE and cost-effectiveness of a cell-based quadrivalent influenza vaccine (QIVc) in preventing influenza-related outcomes in the pediatric population. We identified 16 clinical studies that included data on the VE of a QIVc or the relative VE (rVE) of a QIVc versus an egg-based QIV (QIVe) in children and/or adolescents, 11 of which presented estimates specifically for the pediatric age group. Of these, two studies reported rVE against hospitalizations. Point estimates of rVE varied from 2.1% to 33.0%, with studies reporting significant benefits of using a QIVc against influenza-related, pneumonia, asthma, and all-cause hospitalization. Four studies reported rVE against influenza-related medical encounters, with point estimates against non-strain specific encounters ranging from 3.9% to 18.8% across seasons. One study evaluated rVE against any influenza, with variable results by strain. The other four studies presented VE data against laboratory-confirmed influenza. Three health economics studies focusing on a pediatric population also found the use of QIVc to be cost-effective or cost-saving. Overall, using a QIVc is effective in pediatric patients, with evidence of incremental benefits over using a QIVe in preventing hospitalizations and influenza-related medical encounters in nearly all published studies.

## 1. Introduction

Seasonal influenza continues to place a substantial burden on healthcare systems. In the U.S. alone, influenza caused 9 million–41 million cases, 140,000–710,000 hospitalizations, and 12,000–52,000 deaths annually between 2010 and 2020 [1]. Certain groups, including children < 5 years of age, older adults, and individuals with some chronic medical conditions, are at increased risk of developing severe influenza-related complications. In the U.S., annual vaccination against influenza is recommended for all individuals of >6 months of age [2]. Even so, annual coverage rates continue to fall short of the 70% Healthy People target [3].

Of the four types of influenza virus, influenza A and B cause seasonal epidemics, with influenza A strains also being responsible for pandemics, which occur approximately every 10–50 years [4]. Influenza A viruses are divided into subtypes based on their hemagglutinin (H) and neuraminidase (N) surface proteins, with A/H1N1 and A/H3N2 currently circulating annually. These viruses are continually evolving, with changes in the structures of H and N due to antigenic drift, and therefore require annual vaccine updates to ensure the highest chance of matching with circulating strains [5]. Strains selected for inclusion in seasonal vaccines are generally based on circulating strains detected in the winter season of the alternative hemisphere (e.g., Northern Hemisphere recommendations are based on Southern Hemisphere strains), and, therefore, there is potential for mismatch between vaccine strains and circulating strains once the influenza season begins [6]. The chance for mismatch between circulating and vaccine strains continues and increases throughout the duration of the season, which often ends more than a year after the initial strain selection. This has resulted in poor or no vaccine effectiveness (VE) against certain strains in some previous seasons [7,8,9].

In addition to antigenic drift, the virus may also evolve during the traditional influenza vaccine manufacturing process in chicken eggs. The growth and passage of influenza candidate vaccine viruses (CVVs), used to make conventional seasonal vaccines, in chicken eggs has been associated with reduced VE due to viral egg-adaptive mutations altering the structure of the H and N surface binding proteins [10,11]. Egg adaptation can result in a reduced degree of match between vaccines and circulating viruses, leading to suboptimal immune responses in vaccine recipients. To date, decreased VE due to egg adaption has mostly affected the degree of match to A/H3N2 strains, which have also been associated with the highest levels of severe disease and hospitalization, particularly in older adults [12,13] However, egg adaptation has also been noted in A/H1N1pdm09 strains [14]. Therefore, an alternative method to producing CVVs in eggs has the potential to increase VE.

Cell-based and recombinant influenza vaccines overcome the potential for mismatch due to egg-adaptive mutations. Cell-based vaccines are developed by using Madin–Darby canine kidney (MDCK) cells for the propagation of CVVs instead of chicken eggs, with collected antigens then being purified, inactivated, and tested prior to vaccine shipment [15]. Cell-based production has a number of additional advantages over egg-based production beyond the elimination of egg adaptation. These include the potential for upscaling and rapid manufacturing, the elimination of dependence on a high-quality egg supply from a vulnerable animal population that itself is susceptible to influenza outbreaks and pandemics, and an increased optionality and likelihood of protection as many strains that cause disease in humans cannot grow well in eggs [16]. Cell-based vaccines can also overcome egg allergy concerns, which remain a practical hurdle to achieving higher vaccination rates, albeit not a scientific concern. However, cell-based manufacturing is currently more expensive than egg-based manufacturing, which impacts the cost-effectiveness estimates of cell-based influenza vaccines. QIVc (Flucelvax^®^, CSL Seqirus, Maidenhead, UK), a cell-based quadrivalent influenza vaccine, was approved in the U.S. in 2016 and is currently indicated for all individuals eligible for vaccination (those aged 6 months and older) [17]. In clinical trials, QIVc has been shown to be immunogenic and efficacious with a good safety profile in both children and adults [18,19,20] and elicits comparable antibody titers to egg-based quadrivalent influenza vaccines (QIVe) [21,22,23].

Children are most susceptible to infection by influenza viruses, and young children, particularly those of <2 years of age, are at increased risk of severe complications [24]. In addition, children have been noted to be key vectors of influenza transmission. Therefore, an effective vaccine for this age group can have wider public health impacts by preventing the spread to other vulnerable groups, such as older and immunocompromised adults [25,26]. Immune responses to influenza exposure in early childhood have been shown to shape the response to influenza viruses experienced later in life [27,28] Immunologically naïve children vaccinated with egg-adapted vaccines have been shown to mount antibody responses against egg-adapted epitopes [29], which may influence their ability to effectively respond to circulating influenza strains during adulthood. Cell-based vaccine use may, therefore, not only increase the impacts of vaccination in this age group but also avoid the development of unhelpful immune responses targeting egg-adapted surface proteins. In this review, we provide an overview of the currently available published literature on the efficacy, effectiveness, and cost-effectiveness of QIVc in preventing influenza-related outcomes, with a focus on the pediatric population.

## 2. Methods

### 2.1. Literature Search

We performed a targeted literature review evaluating the VE (both vaccine efficacy and effectiveness) of QIVc across age groups, with a focus on children and adolescents (6 months to 17 years of age). We searched MEDLINE and PubMed using the following search terms: “cIIV4” OR “QIVc” OR “QIVe” OR “quadrivalent egg-based” OR “quadrivalent cell based” AND “vaccine effectiveness against laboratory confirmed influenza” OR “vaccine effectiveness against ILI” OR “vaccine effectiveness against hospitalization” OR “vaccine effectiveness against ED” OR “vaccine effectiveness against death” OR “comparative effectiveness”. A 10-year time frame was used from June 2013 to June 2023. No language restrictions were applied, but English-language publications were prioritized. In addition to published papers and manuscripts, we also reached out to the sponsor for any more recent data that were to be presented at upcoming conferences after the June 2023 cut-off date.

### 2.2. Identification of Relevant Publications

For inclusion in the analysis, publications had to describe original research evaluating the efficacy or effectiveness of QIVc against influenza-like illness, lab-confirmed cases, influenza-related medically attended encounters, hospitalizations, or mortality. Publications describing the VE of other influenza vaccines (e.g., QIVe) were included provided they included a comparison with QIVc. Studies were excluded if they did not report hazard/odds ratios or VE estimates against any of the outcomes mentioned above. Studies based in the U.S. were prioritized, but data from other countries were not excluded. Data on health economic outcomes are presented separately.

### 2.3. Evaluation of Outcomes

Studies are presented based on the outcomes evaluated (i.e., lab-confirmed or any influenza, influenza-related medical encounters, influenza-related hospitalizations/emergency room (ER) visits, and cost-effectiveness). Due to the varying study designs, no formal comparisons were made between VE estimates for QIVc and other vaccines in this analysis, and relative vaccine effectiveness (rVE) estimates versus other influenza/comparator vaccines for any of the listed outcomes are presented only if previously reported in published studies.

## 3. Results

In total, 21 studies were found that reported data on the VE (efficacy or effectiveness) of QIVc. A total of 16 included data on children or adolescents (Table 1): 13 published manuscripts and 3 conference abstracts where no manuscript had yet been published [20,30,31,32,33,34,35,36,37,38,39,40,41,42,43,44]. One study reported vaccine efficacy in children aged 2 to< 18 years [20], one reported effectiveness in children [39], and the other fourteen reported vaccine effectiveness or rVE vs. QIVe in both adults and children [30,31,32,33,34,35,36,37,38,40,41,42,43,44]. Fourteen were performed in the U.S. [30,31,32,33,34,35,36,37,38,39,40,41,42,43], whereas two presented data from other countries [20,44]. Overall, eight reported data from the 2017–2018 influenza season [20,32,33,34,35,36,40,43], seven from 2018–2019 [20,30,31,32,38,41,43], five from 2019–2020 [32,37,39,43,44], and one from 2022–2023 [42]. One study was a randomized clinical trial [20], nine presented the findings of retrospective-cohort- or claims-based studies [30,31,32,33,36,37,38,39,40,41], and the five remaining publications included details of test-negative case–cohort studies [34,35,42,43,44]. A further seven studies presented data on health economic outcomes, which included data on the pediatric population (Table 2) [45,46,47,48,49,50,51].

### 3.1. QIVc in Pediatric Populations

**Figure 1:**
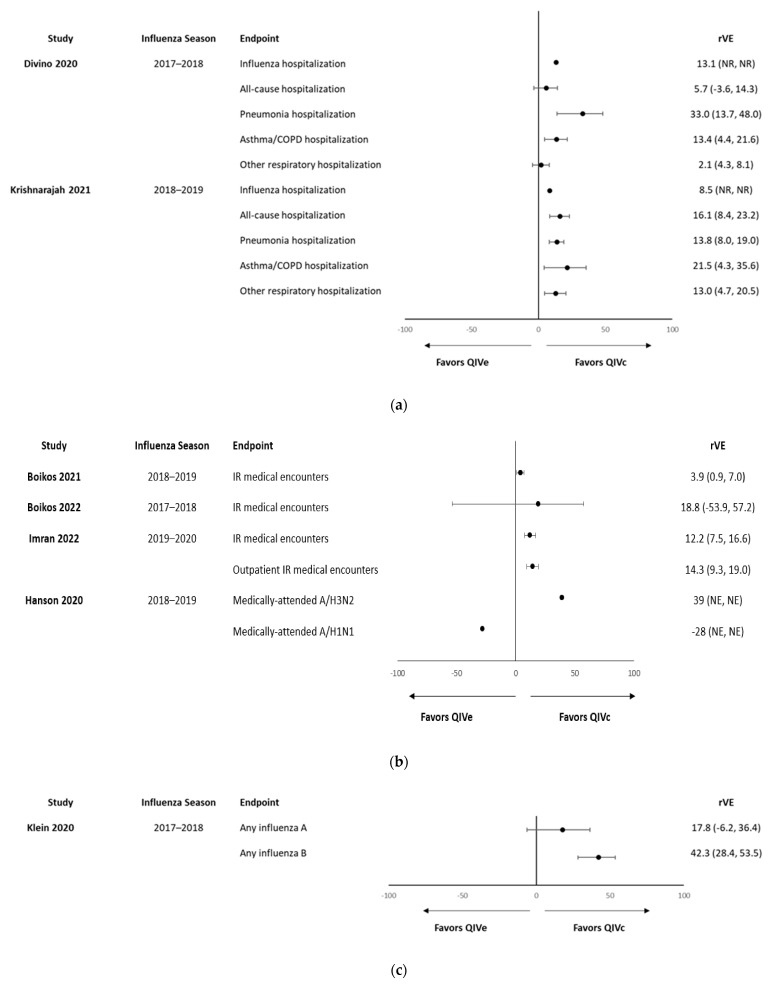
Forest plots of estimates of rVE (95% confidence interval) for QIVc versus QIVe against (**a**) hospitalizations, and (**b**) medically attended and (**c**) laboratory confirmed A or B [30,32,36,38,39,40,41].

In total, 1 study reported the efficacy of QIVc in children [20], and 10 specifically reported VE or rVE data for the pediatric population (Table 1) [30,32,35,36,38,39,40,41,42,44]. The remaining studies provided pooled estimates of VE or rVE across the entire populations evaluated (i.e., children and adults). Of the 10 studies that included specific estimates for pediatric age groups, 3 reported VE [35,42,44], whereas the other 7 reported rVE vs. QIVe against hospitalized or medically attended/any influenza (Figure 1) [30,32,36,38,39,40,41].

### 3.2. Laboratory-Confirmed or Any Influenza

#### 3.2.1. VE

Four studies reported vaccine efficacy or effectiveness against laboratory-confirmed or “any” influenza [20,35,42,44]. In a phase 3 randomized control trial, the efficacy of QIVc against laboratory-confirmed influenza A or B across three influenza seasons was compared with a non-influenza vaccine (meningococcal ACWY) in children and adolescents aged 2–17 years living in eight countries worldwide. Of the 2257 participants who received QIVc, 175 (7.8%) had laboratory-confirmed influenza during the study period, compared with 364 (16.1%) of the 2254 participants who received the control vaccine. The VE was 54.6% (95% confidence interval (CI): 45.7% to 62.1%), with the highest efficacy against A/H1N1 (80.7%; 69.2% to 87.9%), followed by influenza B (47.6%; 34.1% to 60.0%) and A/H3N2 (42.1%; 20.3% to 57.9%). The VE was similar across age groups, with 62.7% (38.1% to 60.2%) in 2–3-year-olds and 53.3% (43.4% to 61.5%) in 4–17-year-olds [20].

The DRIVE study platform included multiple test-negative and cohort studies across Europe during the 2019–2020 influenza season. While the study period was truncated due to the beginning of the COVID-19 pandemic, the VE for QIVc across the studies was 72% (95% CI: −100% to 96%) for the primary-care outcome for children aged 6 months to 17 years but was not estimable in the hospital setting. By comparison, the VE for other vaccines varied from 53% to 81%, but all estimates apart from that for the most commonly used QIVe (Vaxigrip Tetra^®^, Sanofi Pasteur) were confounded by the low numbers of individuals vaccinated, resulting in large confidence intervals [44].

A community cohort study in the U.S. performed during the 2022–2023 season reported the overall VE for seasonal influenza vaccination against influenza A viruses as 71% (31% to 90%) in children aged 1–17 years, adjusted for underlying chronic health conditions and COVID-19 vaccination status. While no specific data were presented for QIVc, approximately 84% of individuals had received QIVc [42].

Finally, in a test-negative case–control study comparing QIVe and QIVc, the overall VE in children was lower for QIVc than the QIVe (QIVc: 36% (12% to 54%); QIVe: 55% (45% to 64%)). The VE in children was also lower for QIVc than QIVe against A/H1N1 (56%; 7% to 15% vs. 99%; 80% to 93%), but it was similar against A/H3N2 (QIVc: 47%; 14% to 67% vs. QIVe: 40%; 21% to 54%) [35].

#### 3.2.2. rVE

One study reported specific rVE estimates against any influenza in pediatric patients. The study, performed during the 2017–2018 season in California, estimated the rVE for QIVc vs. QIVe against influenza A in children aged 4–17 years at 17.8% (−6.2% to 36.4%), with an absolute VE compared with unvaccinated individuals of 36.0% (28.2% to 43.0%). The estimates were higher against influenza B (rVE vs. QIVe: 42.3%; 28.4% to 53.5%; VE 45.8%; 33.7% to 55.8%) [40].

### 3.3. Influenza-Related Medical Encounters

Four studies specifically reported the rVE of QIVc vs. QIVe against influenza-related medical encounters in children and/or adolescents [30,32,38,39]. Firstly, in a retrospective cohort study evaluating influenza-related medical encounters in the U.S. population during the 2018–2019 influenza season, the rVE of QIVc vs. QIVe in the pediatric population (age 4–17 years) was 3.9%, with a 95% CI of 0.9% to 7.0% [30]. In a separate analysis of three retrospective cohort studies in the U.S. over the 2017–2020 influenza seasons, the rVE of QIVc vs. QIVe varied by season, with estimates of 18.8% (95% CI: −53.9% to 57.2%) in 2017–2018, 3.9% (0.9% to 7.0%) in the 2018–2019 season, and 12.2% (7.5% to 16.6%) in the 2019–2020 season for 4–17 year-olds [32].

In a retrospective cohort study evaluating the rVE of QIVc versus QIVe in children and adolescents during the 2019–2020 influenza season in the U.S., fewer influenza-related medical events were reported among the 60,480 QIVc recipients compared with the 1,240,990 QIVe recipients, resulting in an rVE of 12.2% (7.5% to 16.6%) for any influenza-related medical events and 14.3% (9.3% to 19.0%) for outpatient events. Few inpatient events were recorded, and no differences were observed between the vaccines in hospitalized encounters [39].

Finally, a study presented at the 2020 Annual Conference on Vaccinology Research also evaluated the rVE of QIVc compared with QIVe against medically attended influenza. A total of 760 individuals of ≥4 years of age received QIVc, whereas 208 received a QIVe. No significant differences were seen between the vaccines in children aged 4 to 17 years against either A/H1N1pdm09 or A/H3N2 [38].

### 3.4. Influenza-Related Hospitalizations/ER, and Other Hospitalizations

Two studies reported the rVE of QIVc vs. QIVe against influenza-related hospitalizations, ER visits, or other hospitalizations in the pediatric population [36,41].

Firstly, in a retrospective claims-based study of QIVc compared with QIVe during the 2018–2019 season in adults and children living in the U.S., the rVE of QIVc vs. QIVe was 6.5% (0.1% to 12.5%) against influenza-related hospitalizations and ER visits for the overall study population. When evaluated by age group, the rVE was 8.5% for children aged 4–17 years, although this value was not statistically significant (*p* = 0.2664). In this age group, significant benefits of using QIVc were seen in all-cause hospitalizations (rVE: 16.1%; *p* = 0.0001), any respiratory hospitalization/ER visit (rVE: 13.8%; *p* < 0.0001), pneumonia-related hospitalizations/ER visits (rVE: 21.5%; *p* = 0.0165), and asthma/COPD/bronchial hospitalizations/ER visits (rVE: 13.0%; *p* = 0.0026). No differences between the vaccines were observed in rates of urinary tract infection hospitalizations/ER visits (rVE: 3.5%; *p* = 0.6921) [41].

In the second retrospective cohort study evaluating rVE in the 2017–2018 influenza season in the U.S., the rVE against influenza-related hospitalizations/ER visits for the overall study population (≥ 4 years) was higher than in the previous study (14.4%; *p* < 0.0001). However, as with the previous study, no significant difference was seen between the vaccines in the 4–17-year age group (rVE: 13.1%; *p* = 0.1201). In contrast with the 2018–2019 study, there were also no significant differences between QIVc and QIVe in children against all-cause hospitalizations (rVE: 5.7%; *p* = 0.2242) or other respiratory hospitalizations/ER visits (rVE: 2.1%; *p* = 0.5067); however, significant benefits of using QIVc were seen in the rates of pneumonia-related and asthma/COPD/bronchial hospitalizations/ER visits (rVE 33.0%, *p* = 0.0019 and rVE: 13.4%, *p* = 0.0043, respectively) [36].

### 3.5. Cost-Effectiveness of QIVc in Pediatric Populations

A total of seven studies conducted in Argentina, Brazil, Germany, Italy, Spain, Taiwan, and the U.S. that included pediatric age groups in cost-effectiveness analyses were identified (Table 2) [45,46,47,48,49,50,51]. Of these, three studies directly evaluated the cost-effectiveness of QIVc in children and adolescents, whereas the others included pediatric age groups in the overall assessments. Three additional studies also considered the costs of QIVc and QIVe but did not include a formal cost-effectiveness analysis [36,37,41].

One study directly evaluated the cost-effectiveness of QIVc in children aged 6 months to 17 years living in Taiwan. The rVE was estimated as 8.1% during a standard season and 18.8% during an egg-adapted season, based on previously published estimates [45]. The model indicated that the overall burden of influenza would be reduced by 4.5% if QIVc was used instead of QIVe. In a standard season, the incremental cost-effectiveness ratio (ICER) was estimated as USD 68,306 from a payer perspective, considerably below the Taiwan cost-effectiveness threshold of USD 99,177. In an egg-adapted season, which would result in a lower VE for QIVe, the ICER was reduced to USD 45,657 [46].

In a second study evaluating the use of QIVc in Italy, the cost-effectiveness analysis by age group showed that the use of QIVc was the dominant strategy in children aged 6 months–8 years, and 9–17 years, with cost-savings compared with the use of QIVe [47].

In a third study in the U.S., the use of QIVc instead of QIVe in the pediatric population (6 months to 17 years) was shown to be cost-saving in both low- and high-incidence influenza seasons, with savings of USD 468.7 million to USD 1.4 billion from a societal perspective and USD 252.8 million to USD 989.8 million from a payer perspective [48].

The other four studies included pediatric populations in their overall analyses of cost-effectiveness, based on local recommendations and vaccination policies. All studies found the use of QIVc to be cost-effective or cost-saving compared with QIVe [45,49,50,51].

## 4. Discussion

Overall, QIVc was reported as being effective in the pediatric population, and while comparisons with QIVe varied, the rVE in pediatric patients generally favored QIVc in the prevention of hospitalizations and medically attended influenza. While clear benefits of QIVc vs. QIVe were seen against more severe influenza outcomes, only one study directly evaluated rVE against laboratory-confirmed influenza specifically in pediatric populations, with varying results by strain.

Unfortunately, only limited data were available that focused specifically on the VE of QIVc and rVE vs. QIVe in pediatric populations. In the overall pediatric and adult population, significant benefits of using QIVc over QIVe were seen in a three-season study in the U.S., which evaluated laboratory-confirmed outpatient cases, with rVE estimates of 10.0–14.8% across seasons [43]. For medically attended influenza, significant benefits of using QIVc were observed in terms of influenza-related medical encounters in most seasons, although the trends were not significant in a couple of studies [30,31,32,38,39]. Similarly, most studies found significant benefits of using QIVc in terms of reducing hospitalization rates in pediatric populations [36,37,41,44]. Across all studies, the use of QIVc was associated with significantly lower rates of pneumonia-related (point estimates of rVE: 21.5–33.0% across studies) and asthma/COPD/bronchial hospitalizations (point estimates of rVE: 13.0–13.4%) compared with QIVe recipients [36,41]. In the one study that focused on the evaluation of QIVc versus QIVe in the pediatric population only, the rVE against both influenza-related medical events and outpatient-only events was higher for QIVc, with estimates of 12.2% (95% CI: 7.5% to 16.6%) and 14.3% (9.3% to 19.0%), respectively [39]. While clear benefits of using QIVc were seen against medically attended influenza and hospitalizations, few studies evaluated either VE or rVE against influenza infection. Of the estimates available, the rVE was variable by strain, and the VE estimates for QIVc were generally in the same range as the VE of QIVe, with overlapping 95% confidence intervals between VE estimates, or overlapping zero in the case of rVE estimates [35,40,42]. The interpretation of these results is confounded by the small number of studies together with varying seasons, study designs, and study populations; therefore, further evaluations are required to robustly assess the relative impact of QIVc compared with QIVe on influenza infection and mild cases.

While few studies have specifically focused on evaluating the VE of QIVc in pediatric patients, multiple studies have included pediatric patients in their overall assessments of QIVc, with many performing subgroup analyses by age group. While not as robust as specifically designed studies, these subgroup analyses give us an important insight into the impacts of QIVc in the pediatric population. Even so, subgroup analyses can potentially be subject to bias or being underpowered. Data from adult populations generally indicate a higher VE against laboratory-confirmed, medically attended, and hospitalization endpoints for QIVc compared with QIVe in younger adults (aged 18–64 years) [30,31,32,35,36,37,41,52] and no differences between the vaccines in older adults (≥65 years) [32,53,54]. However, the results are also variable across studies, with some reporting contrasting results [8,34,38,40,55].

Part of the variation in both VE and rVE across the published studies may be explained by seasonal differences. As seasonal influenza vaccines are updated annually, the VE can potentially change substantially across seasons, depending on the degree of mismatch with circulating strains [7]. In addition, egg-based vaccines can vary annually in the degree of egg adaptation of the virus strains, which can also increase mismatch with circulating strains, particularly A/H3N2 [10,11]. The use of cell-based manufacturing techniques avoids egg-adaptive mutations, potentially increasing VE, although the degree of potential benefit varies across influenza seasons and vaccine strains [16,56].

As children are the major age group contributing to influenza transmission [57], vaccination of pediatric patients is particularly important, both for individual protection and at a public health level. Vaccination of children has been shown to provide protection against influenza for other vulnerable groups, such as older adults [26]; therefore, maximizing protection in children can have beneficial effects at a population level. Overall, as the manufacture of influenza vaccines using cell-based techniques appears to increase the potential for improved VE compared with traditional egg-based culture, the use of QIVc across age groups could also potentially increase public health benefits. In line with this, cost-effectiveness analysis shows that the use of QIVc in pediatric populations is cost-effective and, in some cases, cost-saving at a societal level, with any increased costs offset by population-level improvements in influenza-related health outcomes and reductions in healthcare service utilization [45,46,47,49,50,51].

One of the limitations of this review is that we did not perform a meta-analysis across studies. As most of the data were from subgroup analyses, we felt that the generalizations across different study designs and populations may have not been robust enough for a formal meta-analysis. In addition, most of the published clinical studies were performed in the U.S. and so may be less representative of findings in other locations. The studies also varied in the age groups included, with few studies including data on children aged 6 months to 4 years. However, the results from the phase 3 study indicated similar efficacy in young children (aged 2–3 years) as in older children and adolescents [20]. While most of the clinical studies were performed in the U.S., all but one of the cost-effectiveness studies were performed in other countries. However, the majority of the cost-effectiveness studies used VE and rVE estimates based on U.S.-based studies as part of their estimates; therefore, we considered these relevant for inclusion in this overall review of the available published data.

Overall, while there are limited data on the impact of QIVc in pediatric populations, the results indicate incremental benefits against medically attended influenza and hospitalizations. The use of QIVc in pediatric populations has been shown to be cost-effective as part of a broader scheme across age groups, and the lack of potential for mismatch due to egg adaption together with manufacturing benefits means that cell-based influenza vaccines can play an important role in population-level protection against influenza.

## Figures and Tables

**Table 1:** Studies included in this review.

Study	Season	Age Group	Type of Study	Location	Outcomes	N Total Study Population	n Pediatric Population (<18 Years) *	rVE Study Population	VE Study Population	rVE Pediatric Population	VE Pediatric Population
Boikos et al., 2020 [33]	2017–2018	≥4 years	Retrospective cohort	U.S.	Influenza-like illness	QIVc: 92,187 QIVe: 1,261,675	QIVc: 7465 QIVe: 404,510	36.2% (26.1–44.1%)	–	–	–
Boikos et al., 2021 [30]	2018–2019	≥4 years	Retrospective cohort	U.S.	Influenza-related medical encounters	QIVc: 2,125,430 QIVe: 8,000,903	QIVc: 78,602 QIVe: 1,628,038	7.6% (95% CI, 6.5–8.6)	–	4–17 years, 3.9% (95% CI, 0.9–7.0)	–
Boikos et al., 2021 [31]	2018–2019	≥4 years with underlying medical conditions	Retrospective cohort	U.S.	Influenza-related medical encounters	QIVc: 471,301 QIVe: 1,641,915	n/a	13.4% (95% CI, 11.4–15.4%)	–	–	–
Boikos et al., 2022 [32]	2017–2018, 2018–2019, and 2019–2020	≥4 years	3 × retrospective cohort studies	U.S.	Influenza-related medical encounters	2017–2018: QIVc: 92,187; QIVe: 1,261,675 2018–2019: QIVc: 2,125,430; QIVe: 8,000,903 2019–2020: QIVc: 1,499,215; QIVe: 4,126,263	2017–2018: QIVc: 7465; QIVe: 404,510 2018–2019: QIVc: 78,602; QIVe: 1,628,038 2019–2020: QIVc: 60,480; QIVe: 1,240,990	2017–2018: 19.3% (9.5% to 28.0%) 2018–2019: 7.6% (6.5% to 8.6%) 2019–2020: 17.2% (15.8% to 18.6%)	–	4–17 years 2017–2018: 18.8% (−53.9% to 57.2%) 2018–2019: 3.9% (0.9% to 7.0%) 2019–2020: 12.2% (7.5% to 16.6%)	–
Bruxvoort et al., 2019 [34]	2017–2018	≥4 years	Test-negative case–control	U.S.	Hospitalization	1186 cases 6946 controls	Hospitalized cases: QIVc: 8 QIVe: 229	rVE < 65 years: 6% (−46 to 39%) rVE ≥ 65 years 43% (−45 to 77%)	–	–	–
De Marcus et al., 2019 [35]	2017–2018	≥6 months	Test-negative case–control	U.S.	Laboratory-confirmed influenza infections	1757 cases 2280 controls	QIVc: 187 QIVe: 703 Unvaccinated: 1383	–	QIVc: 46% (33% to 56%) QIVe: 53% (45% to 60%)	–	QIVc: 36% (12% to 54%) VE QIVe: 55% (45% to 64%)
Divino et al., 2020 [36]	2017–2018	≥4 years	Retrospective cohort	U.S.	Influenza-related hospitalizations/ER visits, all-cause hospitalizations, and serious respiratory hospitalizations/ER visits	QIVc: 555,538 QIVe: 2,528,524	QIVc: ~15,000 QIVe: ~70,000	Influenza-related hospitalizations/ER visits: 14.4% All-cause hospitalizations: 11.8% Pneumonia hospitalization: 4.2% Asthma/COPD/bronchial hospitalizations: 8.3% Other respiratory hospitalizations: 6.9%	–	4–17 years Influenza-related hospitalizations/ER visits: 13.1% All-cause hospitalizations: 5.7% Pneumonia hospitalizations: 33.0% Asthma/COPD/bronchial hospitalizations: 13.4% Other respiratory hospitalizations: 2.1%	–
Divino et al., 2022 [37]	2019–2020	4–64 years	Retrospective claims-based	U.S.	Influenza-related hospitalizations, any respiratory hospitalizations, pneumonia hospitalizations, and ER visits	QIVc: 1,150,134 QIVe: 3,924,819	QIVc: ~28,300 QIVe: ~92,200	Influenza-related hospitalizations: 5.3% Any respiratory hospitalizations: 8.2% Pneumonia hospitalizations: 6.7% Asthma/COPD/bronchial hospitalizations: 7.6%	–	–	–
Imran et al., 2022 [39]	2019–2020	4–17 years	Retrospective cohort	U.S.	Influenza-related medical encounters	QIVc: 60,480 QIVe: 1,240,990	n/a	Any influenza-related medical encounters: 12.2% (7.5–16.6%) Outpatient encounters: 14.3% (9.3–19.0)	–	4–17 years Any influenza-related medical encounters: 12.2% (7.5–16.6%) Outpatient encounters: 14.3% (9.3–19.0)	–
Klein et al., 2020 [40]	2017–2018	4–64 years	Retrospective cohort	U.S.	Any influenza	QIVc: 84,420 TIVe/QIVe: 932,525	QIVc: 43,735 TIVe/QIVe: 40,685	Influenza A: 8.0% (95% CI: –10, 23) Influenza B: 39.6% (CI: 27.9, 49.3)	–	4–18 years Influenza A: 17.8% (−6.2% to 36.4%) Influenza B: 42.3% (28.4% to 53.5%)	–
Krishnarajah et al., 2021 [41]	2018–2019	4–64 years	Retrospective claims-based	U.S.	Hospitalizations/ER visits related to influenza; all-cause hospitalizations; and hospitalizations/ER related to any respiratory condition	QIVc: 669,030 QIVe: 3,062,797	QIVc: ~169,200QIVe: 741,200	Influenza-related hospital/ER visits: 6.5% (0.1–12.5%), All-cause hospitalizations: 7.9% (6.6–9.1%) Hospitalizations/ER visits related to any respiratory event: 7.7% (6.1–9.4%)	–	4–17 years: Influenza-related hospital/ER visits: 8.5% All-cause hospitalizations: 16.1% Hospitalizations/ER visits related to any respiratory event: 13.8% Pneumonia hospitalizations/ER visits: 21.5% Asthma/COPD/bronchial: 13.0%	–
McLean et al., 2023 [42]	2022–2023	6 months–64 years	Test negative case–control and community cohort study	U.S.	Influenza infection	186 vaccinated, 545 total. 84% received cell-based vaccine	223 vaccinated. Breakdown QIVc vs. QIVe not stated	–	Influenza A: 54% (23–73%) A/H3N2: 60% (25–79%)	–	1–17 years: Influenza A: 71% (31–90%)
Nolan et al., 2021 [20]	2017–2018 and 2018–2019	2–17 years	RCT	Australia, the Philippines, Thailand, Estonia, Finland, Lithuania, Poland, and Spain	Laboratory-confirmed influenza	QIVc: 2258 Comparator (MenACWY): 2256	QIVc: 2258	–	VE: 54.6% (45.7% to 62.1%) A/H1N1: 80.7% (69.2 to 87.9) against influenza A/H1N1 A/H3N2: 42.1% (95% CI, 20.3 to 57.9) B: 47.6% (95% CI, 31.4 to 60.0)	–	3 to <18 years: 54.0 (44.8–61.7%) 2 to< 4 years: 62.7 (38.1–77.5%) 4 to <18 years: 53.3% (43.4–61.5%)
Stuurman et al., 2021 [44]	2019–2020	≥6 months, although varies across studies included	Test-negative and population-based cohort study (13 studies in total)	Finland, France, Italy, Romania, Spain, Austria, U.K., and Italy	Primary care and hospitalized influenza	Not stated	n/a	–	–	–	6 months to 17 years: 72% (−100% to 96%)
Hanson et al., 2020 [38]	2018–2019	≥4 years	n/a	U.S.	Medically attended influenza	QIVc: 760 QIVe: 208	n/a	A/H3N2: VE difference 51% (95% CI −6%, 125%) A/H1N1pdm09: VE difference: −7% (95% CI −33%, 22%)	–	4 to 17 years: H3N2: VE difference 39% (NS) A/H1N1pdm09: VE difference: −28% (NS)	–
Stein et al., 2023 [43]	2017–2018, 2018–2019, and 2019–2020	4–64 years	Test-negative case–control claims-based study	U.S.	Outpatient lab-confirmed influenza	QIVc: 31,824 (2017–2018), 33,388 (2018–2019), and 34,398 (2019–2020) QIVe: 28,709 (2017–2018), 29,962 (2018–2019), and 30,508 (2019–2020)	n/a	2017–2018: 14.8% (7.0% to 22.0%) 2018–2019: 12.5% (4.7% to 19.6%) 2019–2020: 10.0% (2.7% to 16.7%)	–	–	–

n/a—not available. * Includes only the individuals aged < 18 years of age included in each of the studies.

**Table 2:** Health economics studies on QIVc that included pediatric age groups.

Study	Age Group	Location	Study Model Type	ICER/QALY	Cost-Effectiveness Threshold	Conclusions
Rizzo et al., 2019 [47]	≥6 months	Italy	Dynamic age-structured SEIR model combined with static decision-tree model	Payer: EUR 8021 Societal: EUR 1428 By age group: 6 months to 8 years: cost-saving 9 to 17 years: cost-saving	EUR 30,000/QALY	Cost-effective overall and cost-saving in children
Ruiz-Aragon et al., 2020 [51]	9 to 64 years	Spain	Static decision-tree model	Payer: EUR 12,852 Societal: cost-saving	EUR 22,000–EUR 25,000/QALY	Cost-effective
Ballalai et al., 2021 [49]	≥6 months	Brazil	Static decision-tree model	Payer: BRL 17,293 Societal: BRL 16,669	BRL 103,599/QALY	Cost-effective
Cai et al., 2021 [50]	≥9 years	Germany	Dynamic age-structured SEIR model combined with static decision-tree model	Payer: cost-saving Societal: cost-saving	EUR 30,000/QALY	Cost-saving
Urueña et al., 2022 [45]	6 months to 64 years (2 to 64 years, high-risk only)	Argentina	Static decision-tree model	Payer: USD 12,214 Societal: cost-saving	USD 29,736.84/QALY	Cost-effective
Chi et al., 2023 [46]	6 months to 17 years	Taiwan	Age-structured static model	Payer: USD 68,306 Societal: USD 40,090	USD 99,177/QALY	Cost-effective
Mould-Quevedo et al. [48]	6 months to 17 years	U.S.	Dynamic age-structured SEIR model	NR	USD 50,000/QALY	Cost-saving

## Data Availability

Not applicable.
